# Social farming at the intersection of spirituality, well-being, and human health: a qualitative inquiry

**DOI:** 10.3389/fpsyg.2025.1655898

**Published:** 2025-12-09

**Authors:** Eliška Hudcová

**Affiliations:** 1 Department of Social Work, Protestant Theological Faculty, Charles University, Prague, Czechia; 2 Department of Social Work, Univerzita Karlova, Prague, Czechia

**Keywords:** social farming, contact with nature, well-being, spirituality, eco-spirituality

## Abstract

It has been reported many times that nature-based connections enhance human well-being, represented by a balanced physical, psychological, and social well-being. Nonetheless, the spiritual dimension is increasingly considered as an integral component of human well-being. Social farming as one of the forms of nature-based interventions represents approach to social work in both urban and rural spaces, rooted in the fields of informal social care, social prevention, supported employment, and community social work. This study examines, using semi-structured in-depth interviews (*N* = 45), the ways social farming influences humans’ spiritual needs, as presented by John Fisher. The interviews indicate that the practice of social farming fosters an environment where human spirituality is expressed across all four dimensions-personal, communal, ecological, and transcendental. Findings suggest that when appropriately facilitated, natural and agricultural environments can cultivate the spiritual dimension of the individual and, consequently, enhance the complexity of human well-being. It can also further bridge the research on eco-spirituality and environmental social work.

## Introduction

This paper examines the relationship between human well-being, spirituality and social farming from the perspective of social workers, social pedagogues, and social farmers. The World Health Organization (WHO) defines human health as a state of complete physical, mental and social well-being (World Health Organization, 1948). For a person to be considered healthy, their physical body should function optimally; they should be in good mental health, and they should maintain quality social relationships. As part of the changes in the perception of human health, the vertical dimension has also been emphasized as the need to relate to something above, behind, or beyond themselves (Gallus, 2023). Therefore, in 1999, at the 52nd WHO Assembly, a proposal was made to supplement the preamble to the Constitution and redefine how to perceive human health. The proposal states that “*Health is a dynamic state of complete physical, mental, spiritual and social well-being, and not merely the absence of disease or infirmity (defect)*” (World Health Organization, 1999, 4). Although this version was vetoed, the importance of the spiritual dimension of a person is increasingly being highlighted (Gray and Coates, 2013; Saad et al., 2017). Spirituality is an internal, individual matter specific to a particular person and has relationship and relatedness, whether to God or to whatever the person considers “ultimate” at its core, as Hodge (2001) states. He characterizes spirituality as a process of finding meaning in life, purpose, and morally satisfying relationships with oneself, others, the World around one, and what the person understands as the ultimate reality. Arrey et al. (2016) write that spirituality involves a personal quest for meaning in life. While religion is culture and historically specific, spirituality raises the search for a sense of purpose, moral principles and performs its universality for human beings (Hutchinson, 2013). Yet, Boynton and Vis (2020) view spirituality as an inner awareness that is universal, inclusive, experienced and attained and varies from person to person. This paper will examine social farming practice through the lens of John Fisher’s model (2010) on four spiritual dimensions: personal, communal, environmental, and transcendental. Social farming is one of the approaches to either social urban or social rural work, anchored in the fields of informal social care, social prevention, supported employment, community-based social work, as well as multifunctional agriculture or environmental sustainability (UNCED, 1992; Organization for Economic Cooperation and Development [OECD], 2003; Wilson, 2007). Two main perspectives dominate in the field, focusing either on participants or on providers of social farming (Moriggi et al., 2020). In a participant framework, the effectiveness in well-being and social reintegration for different target groups is observed, and as a tool for agricultural innovation and income diversification on the provider’s side. In both approaches, the interest comes “*from a cross-section of disciplines like psychology, environmental health, environmental conservation, ecology, horticulture, landscape planning, urban design, leisure and recreation, public health policy, and medicine*” (Bragg in Torquati et al., 2019, 11).

The following contribution aims to answer research questions, such as “How does participation in social farming foster the expression of spirituality and promote well-being and human health?” and focuses on the participant’s perspective. This interest stems from the lack of studies that encompass these thematic areas of human health, well-being, spirituality, and social farming. Thus, the text will proceed from the assumption that spiritual aspects form an essential component of humans and contribute to their full development. Spirituality will be described generally according to John Fisher’s four dimensions model (2010), which will later be used to examine the performance of spirituality on social farms through a qualitative content analysis of 45 in-depth, semi-structured interviews with social farmers, social pedagogues, and social workers. The validity of the research study results is supported by replication logic referring to a particular finding being tried for different sets of people (Hannes et al., 2010). Social farming and the approaches to social work that are based on human contact with nature in both urban and rural settings will be discussed. In the subsequent analysis, spirituality will be presented in the context of social farming and how it contributes to the overall health of the individual. Finally, the question of eco-spirituality, deep ecology and environmental justice will be raised. The theoretical part and discussion of the article are based on English-language indexed journals in the fields of religion, spirituality, social work, psychology and (multifunctional) agriculture. Relevant book publications are used. Some of the expert sources are of Czech origin.

## Conceptual aspects

### Spirituality

Human well-being is sometimes demonstrated as a unity of physical, mental, social, and economic benefits. However, Macnamara (2012) perceives the fullness of human life and good health in the sustainable harmony of the four levels of personality: physical, mental, social and spiritual. Říčan and Janošová (2016, 8) state that spirituality is related to the deepest innermost being and core of the human being*, “with noble feelings and values that we feel as sacred, with beauty, truth, love and goodness, with true wisdom. Through spirituality, a person transcends himself to the cosmos, to all of humanity and nature, and at the same time expresses his true Self, which is in harmony with himself and finds a deeper meaning in his life*.” Canda (2020) mentions that spirituality focuses on the search for a sense of meaning, purpose, morality, and well-being. Sometimes, spirituality is defined as the ability to self-realize (Dudley, 2016). Spiritual questioning and resourcing can offer individuals strength-based resources (Pandya and Samta, 2018). Dimensions of spirituality are diverse, e.g., McCullough and Tsang (2004) remind that of love of others; altruism as a commitment beyond the self with care and service; gratitude as interpersonal interaction that is perceived as beneficial and that reminds us that our power is limited. Other authors add contemplative practice and meeting together during religious social events (McClintock et al., 2016). Canda et al. (2020) add that when the spiritual dimension of people’s lives is ignored, an opportunity to help people construct holistic narratives that accurately fit their experiences may be missed. The potential for complex human development and their full performance may be reduced.

### Spiritual well-being and spiritual needs

Four Domain Model of Spiritual Health and Well-Being as a dynamic state contributes to the operationalization of the concept of spirituality and will be applied in the analysis of the research responses. John Fisher, referring to the work of Moberg (1984) [in Fisher (2010)] in his article, divides spiritual health into four domains, which are presented in the following Table 1. Unlike the previously cited authors, his classification also includes the environmental domain, which is key for nature-based approaches in social work.

**TABLE 1:** Items comprise four domains of spiritual well-being in SHALOM (Fisher, 2010).

Personal	Communal
Sense of identity	Love or the other people
Self-awareness	Forgiveness toward others
Joy in life	Trust between individuals
Inner peace	Respect for others
Meaning in life	Kindness toward other people
**Environmental**	**Transcendental**
Connection with nature	Personal relationship with the divine/god
Awe at a breathtaking view	Worship of the creator
Oneness with nature	Oneness with god
Harmony with the environment	Peace with god
Sense of “magic” in the environment	Prayer life

The acronym SHALOM reveals its two components: Spiritual Health Measure (SHM) and Life Orientation Measure (LOM). The LOM elicits the “ideals” people have for SH in four sets of relationships with Self, others, environment and/or God. The SHM asks people to reflect on “lived experience/how they feel each item reflects their personal experience most of the time” (Fisher, 2010). The domains include (1) Personal level, i.e., a good internal relationship with oneself regarding meaning, purpose, and life values. He sees self-awareness as a driving force and a transcendent aspect of the human spirit in his search for identity and self-esteem. This is followed by (2) the Communal level, which shows the quality and depth of interpersonal relationships, i.e., the bond between the Self and the Other, about morality, culture, or religion. It is manifested by love, forgiveness, trust, hope and faith in humanity. The author mentions (3) the Environmental level, which he identifies with care for the physical and biological aspects, with a sense of respect, awe and the concept of unity with the environment. He calls the fourth dimension (4) Transcendental, which characterizes a person’s relationship to something that is beyond the human level (cosmic force, supreme interest, transcendent reality or God). In this model, spirituality expands to include a relationship with nature, i.e., a space outside of humans that is visible, physical, and tangible.

Suppose all four levels of human well-being (physical, psychological, social, widened spiritual, sometimes supplemented by environmental and aesthetic) are to be in balance. In that case, human needs must also be satisfied, although they change over time and vary according to the roles the individual assumes. Failure to satisfy needs leads to feelings of dissatisfaction, restlessness, a sense of insecurity, lack, deprivation, tension, and displeasure (Výrost and Slaměník, 2008). Considering spirituality as belonging among the higher needs of the well-known classification of Abraham H. Maslow [in Říčan (2010)], he describes them as the need to belong somewhere, to be loved, to be involved; the need for esteem and respect; the need for knowledge; the need for aesthetics; the need for self-realization; the need for transcendence or spiritual needs. At the top of Maslow’s hierarchy of needs is self-actualization, which is the moment when people transcend the boundaries of their ego better realize their possibilities, are more creative, and are more open to experiences outside of space and time (Maslow, 2014). Maslow calls the last need “transcendence,” a deep experience in which a person feels part of a greater whole. The idea of transcendence and belonging to a higher whole are not only carried by the Western paradigm but also appear in concepts of the world as one family (Vasudhaiva Kutumbakam) or as the interconnectedness, interdependence and responsibility of the personal and social self (dharma) in India and in Africa (Ramanathan et al., 2025). Spirituality is often connected to understanding one’s place in the World, as well as our relationships with others and the World. These questions are also explored in logotherapy by psychologist Viktor Frankl, who views a person primarily as an individual searching for meaning, often finding a reason to live or to cope with difficult situations. He finds meaning in both positive experiences and suffering, in losses and one’s failures. The question of meaning must be asked at various moments in our lives (Frankl, 2011), and the search for meaning in life is then crucial for the development of one’s personality. Frankl lists three main directions for fulfilling meaning: (1) we do something, we create, we create a work; (2) a person experiences something - nature, art, loves people; (3) attitudinal values - attitude toward illness, awareness of one’s limitations, and challenging situations (Frankl, 2011). Neither Maslow nor Frankl are traditionally associated with the physical environment and nature in the search for meaning and transcendence. On the contrary, Indian and African spirituality is tied to a natural framework and is closer to the ecological systems theory of social work. In complementarity with Fisher’s model, the promotion of spirituality in social farming will be outlined through the narratives of respondents working on social farms and in nature.

### Social farming

Social farming practice, focused on participants, opens new spaces for experiencing spirituality, which is interpreted as an integral part of human well-being. Social farming represents an intersectional synergy, creating an innovative environment for individuals in challenging life situations to regain their sense of meaning and self-esteem, access new services, fulfill their needs, and strengthen their social functioning (Di Iacovo and O’Connor, 2009). It can also become one of the tools of social work, whether in the fields of social prevention, social care, mental health difficulties, or work integration, promoting individuals’ integration into society and the labor market expanding the portfolio of community social work activities in both rural and urban areas (Borgi et al., 2019). The beneficial effects of nature on humans, resulting from proper interaction, have been noted by evolutionary psychologists in the past (see, e.g., Ulrich, 1983; Kaplan and Kaplan, 1983). Later contributions of natural elements and areas in residential environments to human health and well-being were studied by de Vries (2006) as well as a decade later by Kondo et al. (2018), Shanahan et al. (2019), or Borgi et al. (2019), who identified beneficial areas having a significant impact, such as stimulating physical activity, positive association with attention and mood, enhancing positive social interactions, promoting the healthy development of children, and fostering personal growth. Green exercises have been identified to have broad health benefits by Pretty et al. (2011), including improvements in psychological well-being, generation of physical health benefits (by reducing blood pressure and burning calories), and facilitation of social networking and connectivity. In 2011, Hassing [as cited in Elings (2012)] studied the positive aspects of social farming for participants and identified five main areas of benefit for people. In his scheme, the informality of the farm environment, which is fundamentally different from institutional social and health care, emerges as a central category. He adds the social environment, i.e., the community of people who stay on the farm or visit it, the role model of the farmer, the diversity of concrete and straightforward farming activities, and the green environment. Similarly, Hemingway et al. (2016) led fifteen qualitative interviews with social farm staff and presented findings about the impact of social farms on participants. The staff appreciated the farm environment for facilitating connections, learning, work (doing), involvement of all senses, memorable experiences, fun, adventure, a sense of achievement, autonomy enhancement, and a sense of being and belonging. Comparable results were reported by a team of researchers led by Murray et al. (2019), who highlighted the social environment of the farm, specifically the opportunity to be in a small, informal group, have lunch together, discuss topics, and foster long-term relationships. Specific social farm surroundings creates a place of respect, a sense of value for others, security, structure, stimulation, reflection, independence and understanding of one’s potential. Murray also sees the figure of the farmer as essential, who works together with others, creates a sense of normality and security and provides emotional support to others. Many beneficial elements of social farming for participants are being referred, but not explicitly in connection with the spirituality, transcendence, or spiritual needs. Spirituality in relation to the environment appears as starting point for ecological and responsible behavior (Omoyajowo et al., 2023) which address the climate crisis (Bock, 2024). Or, spirituality becomes the ground of a non-judgmental and open view of the subject of assistance, similarly to the rudiments of social work. But then the idea of the physical natural world as the promoting actor of spirituality appears randomly (Ramanathan et al., 2025). Spirituality closer to nature-based interventions is articulated under the term “soul level” in the paper by Joschko et al. (2023) on nature-based therapy in individuals with mental health disorders. In response to the social and ecological crisis, the COVID-19 pandemic and the related lockdowns, they observed how therapeutic approaches positioned on human-nature contact improve the comprehensive health of patients in selected German healthcare facilities. They evaluated the effectiveness of working in the garden and the park in young patients with psychosomatic problems. The “soul level” (spirituality) is described here as “*a higher function of human being, presence or consciousness of a person*” (Joschko et al., 2023, 8). However, spirituality has not been thematized significantly in the context of social farming so far.

## Methods of data collection and analysis

The study draws on data obtained between 2021 and 2022 from the international SoFarTEAM project^1^, in which the research team investigated the benefits of staying and working on social farms for people who were experiencing a range of difficulties in their lives, including situations of migration and other forcible displacement, physical disability, addictive behavior, mental health problems; advanced in years, situation of young people not in education employment or training; people with reduced intellectual. To diminish the validity threats the appropriateness of the research design, the procedure of data collection, data analysis, the reporting of the findings, the context of research, ethics and the implications of the research were considered (Hannes et al., 2010). The data set comprised 45 interviews with social workers, social educators and farmers working on selected social farms to see a variety of perspectives to gain a deep understanding of the perceived processes within a specific context. 13 interviews were conducted in the Czech Republic (CZ), 13 interviews in Germany (GE), 9 interviews in Ireland (IR), and 10 interviews the Netherlands (NL). A purposive sampling approach and a qualitative research design were employed to select participants based on their expertise in the social farming sector (Murray et al., 2019). A social farm was defined as an agricultural entity that contributes to social integration in a local context with a focus on workplaces, social services and rehabilitation, or education in accordance with the above theory. The main selection criterion for a purposive sample was the identification of the business entity as a social farm with a history of activity from 5 to 30 years. Criteria such as age and gender were not included, because in such a small sample and qualitatively conceived survey they would not bring a representative answer. Only the length of experience in the field and direct work with participants on social farms were considered. Informed consent was obtained from each communication partner, and the ethical level of the research was addressed. The research team modeled the questionnaire protocol for in-depth interviews, which included both closed and open questions and were conducted via Zoom or in person, lasting from 1 to 2 h. A unified structure was chosen because the interviews were conducted in individual project countries and by different researchers over a more extended period. In this way, deviation from the topic was minimized, as was the interviewer’s influence on the interviewer’s quality. The questionnaire protocol provided a uniform method for recording data, replicability of research strategy, and subsequent analysis and comparison of the content of the responses (Hendl, 2005; Hannes et al., 2010; Lune and Berg, 2017). The interview protocol consisted of several parts (e.g., motivation for establishing a social farm; the biography of the social farmer/social worker/social educator, the prevailing model of the social farm, the required support for participants on social farms; relations between workers and farm leaders; conflict prevention; educational goals; technical adaptation of the farm environment). The key questions for this paper are as “What elements of a social farm are important for the spirituality of supported participants? Do participants experience, for example, friendship, good relationships, a relationship to something higher – God, nature or something else?” Although the research was conducted in four European countries, spirituality was perceived as a universal category and a common ground without any connection to religion, understood as the individual’s capacity to experience a transcendent relationship among people, the non-human environment and, for some, God (Canda, 1988). The interviews were transcribed into the native languages of the researchers, who employed an open-coding strategy. The partially processed data were then translated into English, coded collaboratively and associated into thematic frames. The responses were not classified according to the country of origin, as spirituality is understood as a general category, and the work experience with participants on social farms was predominant. The following section presents the findings from the analysis, which applies Fischer’s SHALOM model of spiritual well-being, supplemented by spiritual needs from Frankl and Maslow. The primary method of analysis is based on deductive procedures (Lune and Berg, 2017). To increase the interpretative validity, the citations and verbatim interview excerpts laying out the participants’ view, perceptions, or experiences are displayed in the following section.

## Results: spiritual well-being and spiritual needs on social farms

The appearance of social farms in urban and rural areas represents a piece of greenery that combines productive, therapeutic and socially inclusive aims. It is designed to give attention to vulnerable groups of people, enabling them to work with nature and significantly change the place where participants benefiting from social services usually spend their time. The following paragraphs utilize analytical frames derived from Fisher’s model, which allows for a detailed picture of the various dimensions of spiritual well-being and spiritual needs, approached in basic lines by Maslow and Frankl on social frameworks. Rural and urban social farms are complementary tools for other types of therapies and interventions in social work, which aim to support the social functioning of participants. It is not universal, and the individuality of the recipients must always be considered. On the other hand, nature-based approaches distinctly support spirituality.

### Spirituality on a social farm as an inner relationship with oneself

The agricultural environment, i.e., the landscape of rural space managed by humans or green cultivated spots in cities (such as community gardens and city social farms), provides impetus to stop. It offers anchoring, “rooting,” and the search for meaning of life, purpose and morality. The anchoring and stopping in silence turn inward to a green scenery. Such statements appear repeatedly in respondents’ answers and create a relationship with oneself regarding meaning and life values. Workers assisting people with an addictive past communicate the following:

“*It gives a sense of place; it is a very special relaxing place, and I hope people feel the same way and can really relax here.*”

“Relationship with oneself supports contact with animals, which supports self-confidence, strengthens the feeling of one’s own power.”

For a person who has had a traumatic experience, even a small thing that is often imperceptible can strengthen one’s sense of a transcendent experience, as demonstrated by the answer to the question about the spirituality of a farmer working with a Syrian refugee.

“*Definitely. Of course. Sure, a lot. When he goes to the farm, he strokes his horse and puts his hand on his head. Moreover, he can just saddle him. And then he gets on him and rides. That is something really great.*”

Turning to oneself through contact with animals can help one orient oneself in one’s competencies, support a person in self-realization, strengthen self-belief and open possibilities for experiences that draw on creative energy, which a green environment can enhance. The “personal domain” corresponds to the needs that Maslow places toward the top of his pyramid. Recollection and meditation contribute to the reconstruction of attitudinal values, the understanding of illness, and one’s own limitations, allowing one to gain insight into challenging situations. The inner peace that is enhanced by being in nature also fulfills spiritual needs. A respondent working with refugees says:

“*It is just hope. We do not offer a training position; we offer a life goal. It is always written on the back of our training presentation, and that is my hope.*”

“*Working on a farm has something meditative about it; it fills it with meaning.*”

Elements such as calmness, meditation, meaning, purpose, and inner reflection are revealed repeatedly. Nature experience on the farm returns people to themselves, and caring for animals and plants then gives an empowering impulse, which is crucial for those who feel lost in helplessness and fragility. In this “personal domain” of spirituality, a double level appears, namely the real possibility of experiencing one’s purpose. In the second line, then, the green spaces enable people with limited abilities to undertake meaningful activities and, through them, experience themselves as empowered, strengthened and competent people.

### Spirituality on a social farm, building relationships with other people (social ties)

The next spiritual dimension discovered while staying on a social farm focuses on people-to-people relations. It concerns depth in horizontal relationships and is linked to trust, respect for others, forgiveness, and kindness. The “other” can be represented by a person but also by a community, togetherness, as Fisher mentions. The instrument of the relationship is a common work in which a larger group participates. On a social farm, this community component appears as one of its main benefits. Cooperation, camaraderie, and cohesion are evident in several statements.

“*It gave them self-confidence. (.). Camaraderie and working together are essential. In the end, we are all human.*”

“*Working in the garden promotes good relationships with clients. This also promotes cooperation.*”

The diagram developed by Elings mentions a group based on local proximity, shared values, and common goals, which are one of the main constitutive elements of a social farm. The following answer illustrates this colorful community.

“*It is quite good when there are people with different disabilities in the group, and different people meet each other. People with different problems must get used to each other and learn from each other. [.] And because the group is diverse, everyone can take something away for themselves.*”

The variability of informal green environments, which enable the meetings of different groups of locals and newcomers, gives the social farm added value that is unique in its combination of agriculture and social work. Social farms differ in this way from institutionalized interventions, as they naturally offer social ties with diverse individuals.

“*I like that there is a lot of respect here for the reason why someone comes here and needs help. We are a very diverse group, from people with intellectual disabilities to people with depression. In private, these two groups would not meet, but here, it is a good combination.*”

The commitment to care and solidarity extends not only to people receiving support on social farms but also to their hierarchical relationships among leaders and participants, as the responsibility for one another emerges more naturally. This environment creates situations for learning and imitation more spontaneously because the work appears in logical succession and brings its responsibilities. The individual relies on cooperation and a collective working atmosphere that also bears conflict situations. Social farmers do not focus on disabilities but rather approach people individually, emphasizing their strengths, namely, what they can do. Such an attitude fosters sensitivity to others and their needs, creating mutual responsibility and respect, as well as promoting ethics and care for the sick, the poor, and the suffering. This reciprocal relationship with another person can be expressed by the need to belong, to be loved, and to be involved.

“*They feel that they are not alone in their problems and lives, that they are surrounded by the environment of our gardens and the presence of other clients and employees.*”

Acting toward others is characterized by friendship, which resonates strongly in responses and is classified by the authors as a component of spirituality.

“*[.] and of course friendship! It is a community where you get to know people, where friendships are formed. Some of them met here and spent a lot of time together. They do everything together. And friendships develop not only between residents but also between staff and residents.*”

On social farms, people are not limited to relationships between participants and supporting staff; they also establish social ties with individuals outside the farm, such as those from the village, the city, or with volunteers. This is another element that distinguishes a social farm from a group home, or family of the traditional (residential) facility, as it is based on the strengths of community social work.

“*Because it is a village, everyone knows the farm and knows the clients. When they think someone is in danger, they call us. [.] I think it is also important for people to belong somewhere, not only on the farm but also outside it.*”

Spirituality in this communal form fosters relationships with another person. Eling’s scheme would enrich the category of “social community” and extend beyond it, as it understands belonging, trust, love, and togetherness as an attitude that transcends the human being.

### Spirituality on a social farm as a relationship outside oneself to the cosmos, to god

Spirituality, encompassing both individual spirituality and communal spirituality, is also manifested through one’s relationship with the extra-human reality – the cosmos and nature. This form is divided, according to Fischer’s model, into the transcendental domain of intangible spirituality (cosmos and God) and the environmental domain of spirituality (nature), which is manifested materially and ecologically on the farm. In the following expression, the nature performed on the farm becomes both the goal and the tool of the relationship to a transcendental being. It is the awareness of a larger scale and fascination with something beyond our control which leads to thoughts about the transcendent. In this dimension of spirituality, nature serves as a motivator that emerges from within a person, contributing to the complexity of human well-being.

“*Social farming is a fulfillment of life for me. Until then, I had the feeling that my life was useless. On my journey, I meet people who are believers. We talk about God almost every day. Every day, we realize that what is built on God cannot be bad. We get everything we need.*”

The higher power was specified in some answers with the word God and reflected the Christian orientation of the social farm, where the Christian faith played a significant role for the farm founders. Expressions can be in meditation, singing, and prayers.

“*A closer relationship with God really exists here. People notice it because we also have a chaplain in the home who regularly calls for services held there. And he knows most of the residents personally, regardless of what religion they belong to or whether they no longer practice any religion, and he always has an open ear for the elderly.*”

“One employee is Catholic, the other is Protestant, we talk a lot about faith. It is important to us. Faith is important to us. It is not restrictive, but we always conclude that our path is God’s path.”

The sense of belonging to something greater appeared more frequently in the responses. Vertical relating may be based on the need and experience of transcending everyday experiences and transpersonal experiences. As far as Maslow’s hierarchy of needs, it includes the need for transcendence and deep experiencing, which allows us to share a greater whole. Earlier in the statement, it was given essential meaning to participants being part of something more important.

“*For clients, working on a social farm means contributing to something bigger. Everything they do has meaning. It makes them feel important, useful and needed.*”

The experience of usefulness and being needy is key for participants. People who experience failure in ordinary interactions can be active actors on a social farm. In other words, they are not just recipients of help but also actively contribute to the care of nature and a healthy environment. Thus, they participate in God’s presence in the World. The search for meaning in life is the core of Frankl’s logotherapy. In one of the directions for fulfilling meaning, he writes about experiences of nature, art, love, and work. Nature and work are very tangible and materialized in social farming, helping to “ground” a person, which further contributes to the quality of their lives, their direction, and their understanding of contexts, as Frankl suggests.

“*This work is vital because it pulls people out of their own inner selves and helps them forget about their problems.*”

“*It gives people a sense of control and achievement and strengthens their self-confidence. They achieve success; it is that simple.*”

The last quote is controversial about the first group of manifestations of spirituality, in which nature and agricultural work allow the participants to perceive their inner selves. On the contrary, mechanical activity enables people to forget traumatic life situations and focus attention on goals other than their problems. This process is, in the same vein, enriched by the awareness of something beyond or above the participants.

### Spirituality on a social farm as a relationship to the environment (ecological relationship)

Fisher calls the last domain of human spirituality “environmental” and characterizes it as a wonder at the beauty of nature, as well as a sense of unity and harmony with it. This type of spirituality is based on physical contact with nature (sweeping, plowing, cleaning, grazing, milking). This relationship is primarily abstract, based on observation, wonder, and respect for nature, its beauty, and its power. Both then motivate care.

“*Contact with nature and how does it affect your view of the World? You must take care of nature, and it is beautiful in it. By working on the farm, you create the foundations for this.*”

“*For some, it is like a fairy tale, green grass and views compared to the environment they are used to.*”

The thematization of care for nature alludes to the ecological framework, much like the previous domain of spirituality, which results from both an admiration for nature and the recognition of human dependence on it. Ecology also challenges the anthropocentric assumption that nature exists solely for humans. On the contrary, people are responsible for their behavior toward nature. Their relationship should be equal and symbiotic. Relation to nature subsequently leads to action for protection. This element is crucial in increasing the self-confidence of people who have not experienced success for a long time. A person with a disability, with social and other disadvantages, becomes a significant factor in nature protection, who actively creates a better space for their life. People themselves can do something very concrete and beneficial, which can lead to their empowerment. In the context of disability, nature can also lead to experiences that a healthy person does not have. A social farmer who works with people with reduced intellect says:

“*I think he sees things that I do not see. Nature is around him; nature talks to him, and he reacts to it. I think that the people who come to me are more connected to the elements of nature than we are.*”

“*Seeing small beauties in nature, for example, a beautiful bird, and naming it. Even though the World is very dark now, people can still be amazed.*”

The environmental and spiritual domains, which are linked to the physical and natural environment, can also be observed in the quotes of communication partners in other categories, as will be discussed further. In social farming, this domain is ultimately key yet not explored enough.

## Discussion

This study aimed to investigate the expression of spirituality and the promotion of well-being and human health through participation in social farming. Health is not understood here as the absence of disease or defect but as the dynamic state of complete physical, mental, social and spiritual well-being. Spirituality, with its diverse forms, was presented through the four dimensions model of John Fisher because other models of spirituality (e.g., Arrey et al., 2016) did not include an environmental stance. The results show that contact with nature on a farm raises questions about how people relate to their inner World and how farm settings, but generally purposive contact with nature, can moderate this process. Worlds such as self-confidence, empowerment, and raising competencies through care for animals appeared many times in the narratives. The findings, which address the personal level, align with the higher needs and the meaning emphasized by Frankl and Maslow. The meaning of life enhanced by contact with nature on farms, as reported in Joschko et al. (2023), is notable. They recall, in this regard, the concept of “salutogenesis,” which was raised by Antonovsky (2002) when discussing stress management. The key point of “salutogenesis” is the enhancement of inner peace and meaning in life, as well as a sense of connectedness with nature and a feeling of acceptance from others. Similarly, self-awareness and a sense of a place, relaxation and meditation allow for a slowdown that is healing.

The connectedness with a physical place was often mentioned in the domain of environmental spirituality. In narratives, terms such as magic, beauty, and amazement, along with radically different settings, were pronounced. Working and staying on farms with plants, animals, and other people fosters a connection and a sense of responsibility to nature. When compared to Hassing’s model (Elings, 2012), spirituality would fit into the group of “green environments,” which motivates participants to calm down and stretch into one’s inner Self. Additionally, the “useful and diverse activities” from Hassing’s perspective provide meaning and offer a way to strengthen creativity, making the individual, who would usually be invisible, significant. Thus, categories are not strictly defined but mixed and overlapping. Environmental spirituality, recalled similarly in the research by Bock (2024) and the responsibility for nature may serve as a barrier to the anthropocentric approach and reaction to consumerism, unsustainable economic growth, and materialism, as represented by the “deep ecology” movement. The deep ecology conceptual framework was articulated most notably by Norwegian philosopher Arne Naess in the 1970s (Naess, 1993; Binka, 2008). It challenges the dualism between humanity and the natural World. Instead, deep ecology posits a “relational self”–an extended ecological self that sees all living beings as interconnected within a unified whole. This ethical stance demands humility, respect, and care not only for other humans but for all beings (Naess, 1993). In the context of social farming, this perspective resonates strongly, as participants often report a sense of reconnection with nature that transcends its functional use and instead nurtures a sense of belonging and responsibility. By erasing the duality between humans and the rest of the World, the reification of nature and living beings, which tempts people to destruction, exploitation and use, may be prevented. Analogous findings come from non-Western spiritual observations about one family with all interconnected beings (Ramanathan et al., 2025). In social farming, the interconnectedness of people with the surrounding World is instructive. It becomes apparent even to those who have not yet had the opportunity, due to their disability or living space, to consider the ecological dependencies between individual organisms. On a social farm torn from their previous environment, people with disabilities often have the first chance to perceive these connections and contribute to their protection. From the perspective of deep ecology, it is appropriate for the practice of social work to transfer primary care and responsibility for the environment in which humans live. The environment is not a static space and is not there solely for the benefit of people but is dynamic and requires active advocacy for its needs. Working on a social farm provides a guiding and practical insight, allowing one to defend the interests of animals and plants. Next to deep ecology thoughts, the interest can be found in (feminists) ethics of care, where caring includes more-than-human subjects and non-human objects. Moriggi et al. (2020) focused on Tronto’s five-stage model of caring, where caring about and caring for nature were revealed as the most pertinent in Green Care practices (e.g., social farming). Care for trains the responsibility, interconnectedness and willingness to engage with the betterment of the World.

They also emphasized the importance of care as a process based on reciprocity, mutual deliberation, and learning, which enables more active participation as a care recipient. Care with goes together with Hassink’s social environment (Elings, 2012), facilitation of social networking and connectivity (Pretty et al., 2011), but primarily with Fisher’s communal domain of spirituality as shown in the results from the interviews. The farm environment fosters learning and imitation more naturally. The individual relies on cooperation and a collective working atmosphere. Such an attitude fosters sensitivity to others and their needs, promoting mutual responsibility and respect, as well as ethics and care for the sick, the poor, and the suffering (Dudley, 2016; Ramanathan et al., 2025). This reciprocal relationship with another person can be expressed through the need to belong, to be loved, and to be involved, which Maslow (2014) describes as the upper rungs of his pyramid. Hemingway et al. (2016) wrote about facilitating connections, being, belonging, and joy on social farms. The results of this research demonstrate the importance of camaraderie and teamwork. Fun and camaraderie are not tangible; they can be felt. They can be perceived as something that transcends individuals, leading to a unique and new experience often associated with spirituality. Camaraderie encompasses kindness, forgiveness, trust, and respect toward others. Additionally, friendship and openness to new encounters can help diminish social exclusion as people from diverse settings and backgrounds come together in one place and make a community together (Borgi et al., 2019).

Finally, the respondents from the qualitative study highlighted their relation to spirituality and God in using this term. In literature, the intersection of social farming and spirituality, as a relationship to transcendence, is merely mentioned. The beauty, magic of nature, and fairy tales of greenery that allude to something beyond, behind, or above humans emerge in narratives that assure the transcendent thoughts in humans’ lives. Admiration for nature brings us back to a sense of responsibility and the ethic of care, as well as to what is often referred to as ecological spirituality (eco-spirituality), which stems from a deep respect for nature (Bock, 2024). The starting point of this framework is the romantic-spiritual attitude toward nature, which is described, for example, as “*a strong respect for nature (which can also take the form of worship of Mother Nature). People are part of nature, which gives them many of its gifts, and man should limit his needs so as not to damage the natural world*” (Krajhanzl, 2014, 100). There is a subtle connection to Christian eco-spirituality, illustrated through the symbolism of natural elements revealed in the Bible: The Promised Land of the Israelites, full of life and fruit; Jesus, who marvels at the beauty of nature, and man as a good steward. “*Christian ecological spirituality provides the key to understanding oneself as “dust from the earth” (Genesis 2:7). Man is made up of the elements of the Earth (and the universe), breathes its oxygen and drinks the planet’s waters*” (Sládek, 2019, 67). Therefore, in Christianity, it is impossible to relate to God without being aware of humanity’s physical side, which is composed of the dust of the Earth and is, in this sense, closely related to the considerations of deep ecology. Another context brings catholic theologian Michael Rosenberger, who develops the primary connection with the Earth through gratitude for “*the gift of creation, fear as a retreat from the miracle of creation, maintaining moderation by the needs of other creatures, willingness to sacrifice for the good of creation as a whole, mercy, or compassion for suffering creatures, and finally humility as recognition of one’s finitude and fragility as a creature*” (Rosenberger, 2017, 128). Christian eco-spirituality conceived in this way returns man to his defenselessness, fragility, and fundamental dependence on nature. The situation is thus reversed. Not nature on humans, but humans are entirely dependent on nature, and this awareness is still shallow in society.

All levels of spirituality can be encountered on a social farm, as respondents shared in their answers. Spirituality thus appears in various forms, and farm environments in both urban and rural areas can be one of the contexts that support the development of all dimensions of a person, serving to promote well-being and health. Spirituality is not only manifested in a vertical relationship but can be seen in relationships with others, with nature, and with the Self.

## Limitations

This study offers fundamental insights into the topic of spirituality experienced on a social farm, which contributes to a good state of well-being and health. It is a pilot study without a control group where validity was supported by theory triangulation and frank methodological process. Its focus was on examining different variants of spirituality and how a social farm can foster their development. The study was based on 45 qualitative interviews with farmers, social workers and social educators. It is, therefore, about their perspective and how they view the issue. The interview was not conducted with the participants in the activities on social farms themselves and their perceptions, thoughts and feelings cannot be thus part of interpretation. The interviews were with workers in four European countries. In Ireland, the Netherlands and Germany social farming represents a relatively strong sector in the framework of social work but still, generalizability is limited. Spirituality is understood as a general category. It is not possible to distinguish whether it is perceived differently in Western or Central European countries. The experiences of different target groups of participants were not examined, nor was it determined whether spirituality differs on social farms focused on education, community work, work integration, or rehabilitation. The issue of gender of age were also not taken into consideration. The ethics of care generally concern women more, but this is not specified in the article. There is considerable room for further research in this area. The interviews as research techniques could be supplemented by long-term observations and other ethnographic research methods to allay concerns about descriptive validity of the accounts. Nevertheless, the topic raises other questions and relationships that have not been explored so far.

## Conclusion

This study has explored the multifaceted role of social farming in fostering spiritual well-being and enhancing human health. Drawing on Fisher’s four-domain model of spiritual well-being, the findings illustrate how social farms serve as unique environments where individuals can reconnect with themselves, others, nature, and transcendent realities. The spiritual dimension is revealed gradually, often implicitly, and is frequently discerned between the lines. Spirituality is supported by reflexivity, which can be fostered by natural and agricultural environments and their associated elements. These informal, non-judgmental settings offer a unique space for introspection. Animals do not judge; farmers focus on individuals’ capabilities and strengths, particularly those of people with disadvantages. Such environments allow space for existential questions–about life’s meaning, belonging to a greater whole, and relationships with others–thus contributing to the fullness of human health and being.

The integration of spirituality into social work practice through nature-based interventions highlights the potential of social farming as a complementary approach to conventional care models. While the study offers valuable insights, it also underscores the need for further research, particularly involving the direct voices of participants, exploring diverse cultural and gender perspectives, and employing longitudinal or ethnographic methods. Future studies could deepen our understanding of how spiritual experiences on social farms contribute to long-term well-being, help overcome crises or natural disasters, and support and social resilience.

## Data Availability

The raw data supporting the conclusions of this article will be made available by the authors, without undue reservation.

## Footnotes

1 Project funded by the Erasmus + program under the reg. no. 2022-1-CZ01-KA210-ADU-000083310 The project partners were the Faculty of Agriculture and Technology of the University of South Bohemia (Czech Republic), Jabok – Higher Vocational School of Social Pedagogy and Theology (Czech Republic), Leitrim Development Company (Ireland), Thuringer Ököherz (Germany), University of Neubrandenburg (Germany) and Wageningen University (Netherlands). More about the project can be found here: Project summary SoFarTEAM - sofaredu.
